# Influence of a repeated peripheral electrical stimulation on substance P and cortisol concentrations, and behavior in German Simmental calves — a pilot study

**DOI:** 10.3389/fvets.2026.1752497

**Published:** 2026-01-30

**Authors:** Theresa Tschoner, Hannah Kerber, Yury Zablotski, Gabriela Knubben-Schweizer, Melanie Feist

**Affiliations:** Clinic for Ruminants with Ambulatory and Herd Health Services at the Centre for Clinical Veterinary Medicine, LMU Munich, Munich, Germany

**Keywords:** analgesia, ethogram, Fleckvieh, nociception, pain

## Abstract

**Objective:**

The aim was to assess plasma substance P (PSPC) and plasma cortisol (PCC) concentrations, vital signs, behavioral parameters, and activity in calves submitted to either an electrical, or a sham stimulus. The hypothesis of this pilot study was that an electrical stimulus increases plasma substance P concentrations in calves due to nociception.

**Methods:**

A total of 24 male calves (43.9 ± 2.0 days old) were included in this study. Calves in PAIN (*n* = 12) were submitted to 5 consecutive electrical stimuli, and calves in CON (*n* = 12) to 5 consecutive sham stimuli. Blood samples to measure PSPC and PCC were taken before (baseline and at 0 min), during (5 min to 25 min), and after (30 min to 7 hours) stimulation. Vital signs and behavior were recorded for 30 min during the stimulation and additionally before each blood sampling time. Activity was assessed over 24 h.

**Results:**

There were no significant differences in PSPC between groups. In PAIN, PSPC were significantly lower at 25 min compared with at baseline and at 0 min. PCC were significantly lower at 4.5 h compared with at baseline and at 0 min in CON. Calves in PAIN showed a significantly lower number of “ear movements,” but a significantly higher number of “shaking of the legs” during stimulation. Calves in PAIN showed significantly more occurrences of “head held below dorsal line” during stimulation, and significantly more occurrences of “eye lids half closed” during and after stimulation, compared with CON. Activity did not differ between groups.

**Conclusion:**

Administration of an electrical stimulus resulted in a decrease of PSPC compared to control animals, despite animals in PAIN showing behavior indicative of nociception. These results may affect the use of substance P as an objective biomarker for nociception for the assessment of pain in cattle from a stimulus that does not cause either tissue damage or inflammation.

## 1 Introduction

Cattle strongly mask signs of pain and do not show obvious pain behavior (1), which makes pain recognition difficult (2, 3) and has often lead to the assumption that cattle are insensitive to pain (1). Therefore, pain as well as pain management in cattle remain a major welfare problem in veterinary medicine (4, 5).

One parameter used to evaluate nociception in cattle and to differentiate between distress caused by nociception and stress is substance P (SP) (6, 7). SP is a neurotransmitter involved in processing noxious information to the brain (8), and a neuromodulator of pain (9). It is synthesized in ribosomes as a prepropeptide and transported via axons to the nerve ends. Following a noxious stimulation, SP is released from the neurons of the spinal ganglion and can be found in afferent neurons of the dorsal horn of the spinal cord, in cells of the dorsal ganglion, and in the dorsal roots of spinal nerves (10). SP is released slowly, with a delayed onset of excitation of the dorsal horn of 20 to 40 s, and a slow response which lasts 30 to 90 s (11–13).

In 2008, Coetzee et al. found that SP concentrations in surgically and sham castrated calves differed significantly, contrary to cortisol concentrations (6). Since then, SP concentrations have been described in cattle undergoing different painful procedures and conditions. However, studies provide conflicting results about the suitability of SP as a biomarker for nociception. SP concentrations were significantly lower in meloxicam-treated compared with placebo-treated calves following castration (14) and scoop dehorning (15). Other studies found no effect of flunixine meglumine on SP following castration (16) or cautery dehorning (17).

The major limitation of using SP as a biomarker for nociception is that research in experimental animals showed that SP controls the migration of inflammatory cells to an inflammatory site (10) and is an inflammatory marker (18). Local injury and tissue damage result in plasma extravasation due to increased plasma permeability, resulting in a release of SP from sensory nerve endings as an inflammatory mediator (19). Recent research found a positive and significant correlation between SP and leucocyte count in calves (20). In animal models, SP is also released in the course of a stress response, and SP and Neurokinin (NK)-1 receptors are located in those neurons of the neuroaxis which are part of the integration of pain, stress, and anxiety (8). The majority of studies in bovine medicine assessed SP concentrations following a painful stimulation resulting in tissue damage, and therefore, local inflammation (6) or during conditions involving the immune system (21, 22). Basic scientific research about the influence of nociception without tissue damage, stress, or inflammation without nociception on SP concentrations in cattle has not been done so far. This study is part of a project with the purpose to evaluate the influence of these stimuli on SP concentrations in cattle. A companion study assessing the influence of stress on PSPC was recently published (23).

The subject of this pilot study was to investigate the effect of nociception without tissue damage on SP. The objectives of the present study were to (1) evaluate the plasma substance P concentrations (PSPC) in calves experiencing a repeated electrical stimulation compared to a control group, (2) describe plasma cortisol concentrations (PCC), and (3) assess behavioral changes during and after the stimulation.

The primary motivation for the present study was to describe PSPC following a nociceptive stimulus not resulting in tissue damage, to distinguish between nociception associated with tissue damage, as described for castration (6) or inflammation such as metritis (21), and nociception occurring in the absence of tissue damage. The hypothesis of the present study was that a noninvasive acute peripheral electrical stimulation results in an increase of substance P, but not cortisol concentrations. This experiment is important, as until now, no biomarker for the assessment of only nociception has been described in cattle and basic research work about SP, which has been used as a biomarker for mostly nociception in cattle, is rare. The results of this study could help other researchers when planning studies including the assessment of SP.

## 2 Materials and methods

All experimental procedures in the present study were approved by the ethics committee of the government of Upper Bavaria (reference number 55.2-2532-Vet_02_20–176). The present study is a pilot study with 12 animals included per group (24–26). Sample collection was done from May 2023 to May 2024.

### 2.1 Animals and group assignment

A total of 24 male German Simmental calves were included in this study. Animals were bought from a market in Miesbach, Bavaria (Germany). On the day of purchase, mean age and weight of calves were 32 ± 2 (28 to 36) days and 80.0 ± 5.6 (68 to 88) kg of body weight. Upon arrival at the Clinic for Ruminants with Ambulatory and Herd Health Services, all animals were submitted to a clinical examination. Blood was taken via puncture of the jugular vein, and a laboratory blood analysis for hematology and blood chemistry was done. Furthermore, 22 out of 24 animals were treated with a live vaccine for bovine respiratory syncytial virus (BRSV) and bovine parainfluenza-3 (PI3) infection and with vitamin E and selenium (2–4 mL/calf s.c., Vitamin E + Selen, Bela-Pharm gmbH & Co. KG, Vechta, Germany) on the day of arrival, and with diclazuril (1 mg/kg BW orally, Diacox® 2.5 mg/mL, Virbac Tierarzneimittel GmbH, Bad Oldesloe, Germany) on the following day. Animals 1 and 2 did not receive this treatment, due the medication only being implemented after these two calves were diagnosed with diarrhea and/or bronchopneumonia during the acclimatization period. To be included in the study, animals had to be clinically healthy on the day before and on the trial day with leucocyte count within the reference ranges of our clinic. Exclusion criteria were acute or chronic diseases and treatment with an antibiotic or NSAID within 48 h prior to the trial.

The study was conducted as a randomized controlled trial, calves were randomly assigned to either control group (CON, *n* = 12) or trial group (PAIN, *n* = 12). Randomization consisted of a lottery with equal numbers of lots for CON and PAIN in sealed envelopes. On the day before each trial, one envelope was randomly chosen by one of two authors (TT, MF) for group assignment. Due to the study setting, researchers knew the group assignment of the animals during the trial.

### 2.2 Housing and husbandry

Calves were housed in individual igloos with visual and hearing contact to other calves. Calves were kept on straw with ad libitum access to water, hay, concentrates, and mineral licks. Calves were fed with whole milk for 3 times per day (three liters each at 07:00 a.m., 12:00 p.m., 07:00 p.m.), except for two calves (1, 2), which were fed with two liters at each feeding, and additionally at 10:00 a.m., 03:00 p.m., and 11:00 p.m. due to feeding management at their farm of origin. A clinical examination was performed in all animals at least once daily by a veterinarian (TT, HK).

### 2.3 Acclimatization period and crush training

Upon request of the Government of Upper Bavaria, calves had an acclimatization period of at least 7 days (12.5 ± 1.5, 8 to 15 days) until the day prior to the day of the trial to get used to the surroundings of the clinic and the veterinarians working with the calves to limit stress for the animals. During that time, all calves were trained to stand in a crush for a total of 7 days, to reduce the influence of stress on the study results. Duration of crush training was 6.3 ± 0.8 (5 to 7) days, due to sickness of either calves or researchers. Calves were brought into an examination room and put into the crush. The head was fixed with a halter and a rope. After trimming the area around the coronary band of the right hind limb, two adhesive surface electrodes (Disposable Adhesive Surface Electrode, Spes Medica Srl, Genova, Italy) were fixed to the trimmed and degreased skin one cm above the coronary band of the lateral and medial claw of the right hind limb each day. Calves were kept in the crush for the duration of 30 min with the electrodes attached.

On the day before the trial day, calves were sedated with xylazine hydrochloride (0.2 mg/kg BM intramuscularly; XYLAZIN 2% Bernburg®, Serumwerk Bernburg, DE). Following local infiltration of the skin with 2 mL procaine hydrochloride (Procasel-2%, Selectavet, Germany), a 16-gauge x 15 cm catheter with an attached extension (PUR Infusionskatheter, Walter Veterinär – Instrumente e. K., Baruth/Mark, Germany) was placed in the left jugular vein. Blood samples for assessment of PCV, hemoglobin, leucocyte count, total protein, and glucose were taken. All animals were clinically healthy and leucocyte count was 5.9 ± 1.2 (4.2–9.9) x10^3^/μl (reference range 4–10 ×10^3^/μl). Findings of blood analysis are given in Supplementary Table 1.

### 2.4 Experimental setup

On the day of the trial, blood sampling was done at 08:00 a.m., followed by a clinical examination. At 08:30 a.m., calves were brought into the examination room and put into the crush. At 08:45 a.m., two surface electrodes were attached as described for the training days. For better attachment, electrodes were additionally wrapped with Fixomull (Fixomull® stretch, BSB medical GmbH, Hamburg, Germany). From 09:05 a.m. to 09:25, electrical or sham stimulation was performed. The electrical stimulation was not intended to model commercial handling procedures but served as a controlled experimental tool. At 09:35 a.m., animals were brought back to their igloos, were they remained until the end of the trial.

### 2.5 Electrical and sham stimulation

Calves in PAIN were submitted to an electrical stimulation using a commercial nerve stimulator (programmable Isolated High Power Stimulator 4,100, A-M Systems, Science Products GmbH, Hofheim, Germany), which has been used for electrical nociceptive stimulation in cattle (27) but not calves before. The stimulus consisted of a constant voltage ramp, with a frequency of 1/60.1 s (16.6 mHZ). The current adjusted automatically to maintain the voltage requested, starting at 1 V, and increasing to a maximum of 50 V within 60 s. Voltage at termination was calculated under this assumption, as the stimulator did not display the voltage at any time point. The stimulus was triggered manually and either applied for 60 s, or until the calf showed a continuous (≥ 5 s) withdrawal response. Stimulation was done five times with an interval of 5 min (09:05 a.m. to 09:25 a.m., stimulus 1 to 5) between the individual stimuli to imitate a painful surgical stimulation (28).

**Figure 1:**
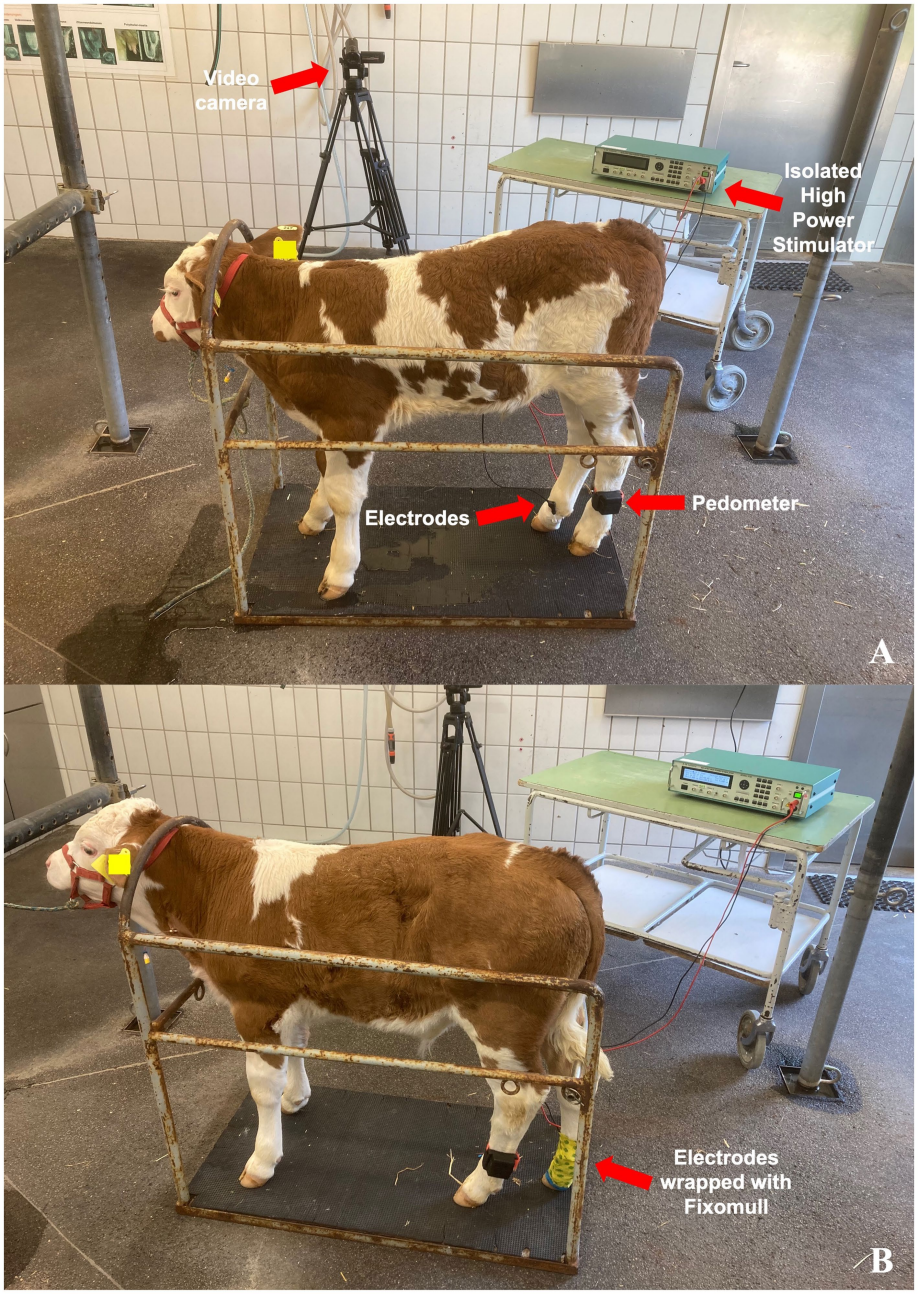
Experimental setup for the assessment of the effect of an electrical stimulus on substance P and cortisol concentrations, as well as behavioral parameters and activity in healthy German Simmental calves. The setup and handling were the same for all calves. Calves in the control group (**A**, CON, *n* = 12) were submitted to 5 sham stimuli (touching of electrodes) every 5 min from 09:05 a.m. to 09:25 a.m. Calves in the trial group (**B**, PAIN, *n* = 12) were submitted to 5 electrical stimuli for a duration of up to 60 seconds, with the voltage increasing from 0 to 50 Volt.

Animals in CON were submitted to the same experimental setup as animals in PAIN (Figure 1) but without the electrical stimulation. The effect of the electrical stimulation was simulated by a person lightly touching both electrodes for a duration of 60 s at each stimulation (sham stimulus).

### 2.6 Collection of blood samples

Schedule of collection of blood samples is presented in Table 1. Samples are referred to as time from the baseline in minutes and hours. Before each blood sampling, the intravenous catheter was flushed with 5 mL of 0.9% saline, then flushed with blood for three times, and 5 mL of blood were discarded. Blood samples were then taken with a new syringe, and the catheter was flushed again. Blood samples for assessment of PSPC and PCC were transferred to 2 mL EDTA (EDTA 3KE, Sarstedt AG & Co. KG, Nuremberg, Germany) tubes right after sampling, kept on ice, and brought to the laboratory of the Clinic for Ruminants with Ambulatory and Herd Health Services. EDTA tubes for SP samples were spiked with 9 μL aprotinin per tube and were always kept in a refrigerator or on ice. All samples were centrifuged within 2 h after blood collection (4 °C, 1600 x g for 15 min). Blood plasma was kept at −80 °C until analysis.

**Table 1:** Schedule of blood sampling to assess substance P and cortisol concentrations in *n* = 24 German Simmental calves submitted to either an electrical or a sham stimulus (indicated by box).

Time	Sample	Procedure
08:00 a.m.	Baseline	Baseline, in igloo
09:00 a.m.	0 min	Standing in crush
09.05 a.m.	5 min	Electrical Stimulus 1
09:10 a.m.	10 min	Electrical Stimulus 2
09:15 a.m.	15 min	Electrical Stimulus 3
09:20 a.m.	20 min	Electrical Stimulus 4
09:25 a.m.	25 min	Electrical Stimulus 5
09:30 a.m.	30 min	Standing in crush
09:35 a.m.	35 min	After being brought back to igloo
09:45 a.m.	45 min	
10:00 a.m.	1 h	In igloo
10:30 a.m.	1.5 h	
11:00 a.m.	2 h	
12:00 p.m.	3 h	
13:30 p.m.	4.5 h	
16:00 p.m.	7 h	

Samples were taken minutes (min) and hours (h) following a baseline sample collected 1 h before the intervention started.

### 2.7 Cortisol and substance P analysis

ELISA Kits (ENZO®, Enzo Life Sciences GmbH, DE) were used for determination of PSPC (29, 30) and PCC (29). Optical densities were determined in duplicate, and means were used for the calculation of concentrations. Lower and upper limits of quantification for the SP ELISA Kit were 9.8 pg/mL and 9,687.6 pg/mL. Sensitivity for SP was 102.3 pg/mL. The intra- and interassay coefficient of variation was calculated to be 15%, and 26% for samples being measured repeatedly. Lower and upper limits of quantification for the cortisol ELISA Kit were 153.0 pg/mL and 9,919.0 pg/mL. Sensitivity for cortisol was 501.9 pg/mL. The intra- and interassay coefficient of variation was calculated to be 15%, and 40% for samples being measured repeatedly.

### 2.8 Assessment of vital signs and behavioral scoring

Assessment of vital signs (heart rate (HR), respiratory rate (RR)) and behavioral scoring was always done by one of two authors (HK was supervised by TT) at all sampling times before blood sampling. Behavioral scoring was done using an ethogram including evaluation of occurrences (“yes,” “no”) of behavior of the animal, position of the head, dorsal line of the back, and the facial grimace scale (expression of eyes, nose, and ears) (Table 2) (3, 4). Due to low counts per time point, we collapsed the 12 time points into three intervals to increase statistical power, stabilize estimates, and improve precision and model convergence. Occurrences of behavior were assessed before stimulation (Baseline, T0), during (5 min to 25 min) and after (30 min to 7 hours) stimulation.

**Table 2:** Parameters to assess behavior in calves before, during, and after the administration of either a sham or an electrical stimulus to evaluate course of plasma substance P and cortisol concentrations, as well as thebehavior and the activity in calves modified as described by Feist et al. (4) and Gleerup et al. (3).

Parameter	Present	Timing															
		Baseline	0 min	5 min	10 min	15 min	20 min	25 min	30 min	35 min	45 min	1 h	1.5 h	2 h	3 h	4.5 h	7 h
Heart rate																	
Heart Rate (beats per minute)	–																
Respiratory rate																	
Respiratory Rate (Breath per minute, no value if sniffing)	–																
Behavior of animal (head)																	
Looks at observer, head held high, ears to front	Yes/No																
Looks at observer, ears back	Yes/No																
Does not look at observer, head down, ears back	Yes/No																
Position of head																	
Head held high, physiologic position	Yes/No																
Same height as dorsal line	Yes/No																
Below dorsal line	Yes/No																
Dorsal line																	
Straight, physiologic	Yes/No																
Mildly arched	Yes/No																
Severely arched	Yes/No																
Eyes																	
Normal, wide eyes	Yes/No																
Lids half closed	Yes/No																
Eyes closed	Yes/No																
Dropping eyelids	Yes/No																
Eyes wide open	Yes/No																
Eyes popping out of head	Yes/No																
Dull, staring eyes	Yes/No																
Rotating bulb, nystagmus	Yes/No																
Nose																	
Clean, wet nose	Yes/No																
Dry nose	Yes/No																
Wide nostrils	Yes/No																
Unclean, „dirty “nose	Yes/No																
Wrinkling of skin at back of nose	Yes/No																
Ears																	
Ears held in normal position, facing front	Yes/No																
Both ears facing back	Yes/No																
Frequent moving of ears	Yes/No																
Ears low	Yes/No																
Reduced reaction to noise	Yes/No																

Parameters were assessed at each blood sampling time.

During either electrical or sham stimulation (09:00 a.m. to 09:30 a.m., 0 min to 30 min), behavior of the calves was recorded using a video camera to assess frequency of different parameters [movement of the ears and head, kicking or shaking of the legs, tail movements, moving forwards and backwards in crush, vocalization, teeth grinding, urinating, or defecation (Table 3)]. Frequency of behavior was assessed in total over 30 min, as the duration of the electrical stimulus was not consistent for all PAIN calves but terminated when calves showed a continuous (≥ 5 s) withdrawal response. Video recordings were always evaluated by the same person (TT).

**Table 3:** Parameters to assess behavior in calves before, during, and after the administration of either a sham or an electrical stimulus to evaluate course of plasma substance P and cortisol concentrations, as well as thebehavior and the activity in calves.

Behavioral parameter	Description of parameter	n/30 min
Ear movements	Movement of both ears or of one individual ear; one ear movement was counted as movement to the back and then the front again.	
Movement of the head	Head shaking or banging.	
Kicking with Hind Legs	Kicking with one or both hind legs as a reaction to either the (painful) stimulus or as evasive behavior.	
Shaking of Hind Legs	Shaking of one or both hind legs as a reaction to either the (painful) stimulus or as evasive behavior.	
Tail Movements	Quick movements of the tail from one side to the other.	
Moving forwards or backwards in crush	Evasive movements in the crush (either to the front or backwards)	
Vocalization	Groaning, moaning, mooing, or other forms of vocalization	
Teeth Grinding	As a reaction to either pain or stress	
Urination		
Defecation		

Behavioral parameters were recorded with a video camera for the duration of 30 min on the day of the trial (from 09:00 a.m. (0 min) until after blood sampling at 09:30 a.m. (30 min)). The different parameters were assessed as frequency (n) in 30 min.

### 2.9 Activity and number of steps

Activity and number of steps was recorded using pedometers and a software for analysis (RumiWatch Systems®, Itin and Hoch GmbH, Liestal, Switzerland). These pedometers continuously record lying, standing, and walking activity, including number of steps (29). All calves were fitted with a pedometer at 08:00 a.m. on the day of the trial. Pedometers were attached on the left hind limb proximal to the fetlock joint. Data recording was done for 24 h.

### 2.10 Statistical analysis

Data analysis was performed using R 4.4.0 (2024-04-24) statistical software. PCC were compared between training days in the crush by simple linear model because data was normally distributed and the variance between days was similar. Robust linear mixed-effects model was used to compare “seconds till termination of electrical stimulus” between different timepoints. Linear mixed effects models were used to study PSPC, PCC, HR, and RR. The predictors “time” (baseline and 0 min:4.5 h) and “group” (control vs. pain) were used as fixed effects with an interaction between them and with a random effect of an individual animal due to the repeated measures of every animal over time. Normality and homoscedasticity of residuals were assessed via visual residual-diagnostics after the models fit. Due to the not-normally distributed and heteroskedastic residuals data for PSPC and PCC were log-transformed. The generalized linear and robust linear mixed-effects models were conducted for every analysis and compared via Akaike’s Information Criterion (AIC) and R^2^. The best model (higher R^2^ and lower AIC) was used for further analysis and post-hoc tests where timepoints and groups were compared. The correlation between PSPC and PCC (across all groups – CON and PAIN – and all timepoints) was assessed using Spearman’s correlation on logarithmically transformed data (both PSPC and PCC were logged). The differences in behavioral and activity parameters between unpaired control and pain groups were assessed via the unpaired two sample t-test in case of normally distributed data and via Wilcoxon rank sum (Mann–Whitney U) test in case of not normally distributed data. Association between groups for categorical behavior and activity predictors were studied via Fisher’s exact tests.

Results with a *p*-value < 0.05 were considered statistically significant. Due to the exploratory approach of the study and small sample size, correction of the *p* values for multiple comparisons was not performed, to decrease the probability of Type 2 error (missing a discovery).

## 3 Results

### 3.1 Electrical stimulation

Duration until and voltage at termination of painful stimulation are given in Table 4. Seconds until termination of stimulus decreased significantly from stimulus 1 to stimulus 4 (*p* = 0.0071) and stimulus 5 (*p* = 0.0054). After removal of the electrodes, no tissue damage or alterations were recorded macroscopically via adspection or palpation.

**Table 4:** Duration in seconds until and voltage (V) at termination of electrical stimulation in 12 German Simmental calves (PAIN), which were submitted to 5 consecutive electrical stimulations.

Stimulus	Termination (seconds)	Voltage (V)
Stimulus 1 (5 min)	48.8 ± 14.4 (16–61)	40.7
Stimulus 2 (10 min)	45.6 ± 15.1 (17–61)	38.0
Stimulus 3 (15 min)	43.6 ± 15.1 (13–61)	36.3
Stimulus 4 (20 min)	41.9 ± 13.3 (17–61)	34.9
Stimulus 5 (25 min)	42.0 ± 12.4 (22–61)	35.0

Range of seconds until termination is given in brackets. Voltage was calculated under the assumption that the voltage increased from 1 V to 50 V within 60 seconds due to the stimulator not displaying voltage at anytime point.

### 3.2 Plasma substance P concentrations

**Figure 2:**
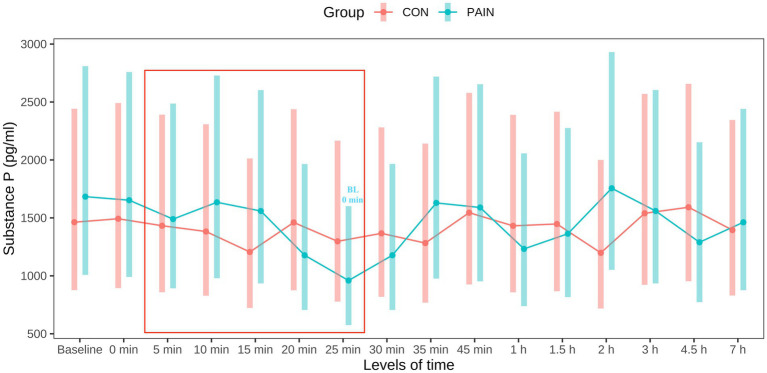
Course of plasma substance P concentrations in calves in CON (*n* = 12, sham stimulus, in red) and PAIN (*n* = 12, electrical stimulus, in blue). Points represent mean values with bars representing the 95% confidence intervals. Baseline blood samples were taken at the igloo 60 min (min) before intervention. Sham/electrical stimulus was submitted every 5 min from 09:05 a.m. (5 min) to 09:25 a.m. (25 min) (red box). From 09:35 a.m. (35 min) onwards, animals were back in their igloos. Significant differences (*p* <0.05) within groups compared with baseline and 0 min are indicated either in red (CON) or blue (PAIN). There were no significant differences between groups.

One sample (CON) was not brought to the laboratory in time and is therefore missing. There were no significant differences between groups at any time point (Figure 2). At baseline, mean PSPC (with lower and upper CI) were 1,465.6 (880.1–2,440.6) pg/ml in CON and 1,685.8 (1,012.3 – 2,807.4) pg/ml in PAIN. Contrary to CON, PSPC in PAIN decreased following the electrical stimulus, and were significantly lower at 25 min compared with baseline (*p* = 0.0057) and 0 min (*p* = 0.0075). After the last stimulus, PSPC in PAIN showed an increase. Mean PSPC concentrations are presented in Supplementary Table 2, and significant differences within CON and PAIN in Supplementary Table 3.

### 3.3 Plasma cortisol concentrations

**Figure 3:**
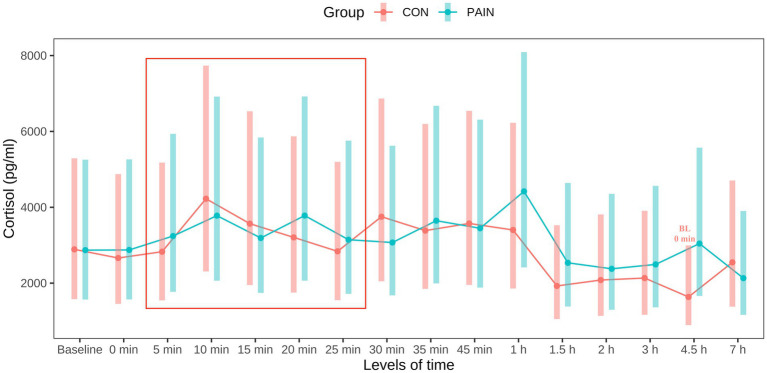
Course of plasma cortisol concentrations in calves in CON (*n* = 12, sham stimulus, in red) and PAIN (*n* = 12, electrical stimulus, in blue). Points represent mean values with bars representing the 95% confidence intervals. Baseline blood samples were taken at the igloo 60 min (min) before intervention. Sham/electrical stimulus was submitted every 5 min from 09:05 a.m. (5 min) to 09:25 a.m. (25 min) (red box). From 09:35 a.m. (35 min) onwards, animals were back in their igloos. Significant differences (*p* < 0.05) within groups compared with baseline (BL) and 0 min are indicated eitherin red (CON) or blue (PAIN). There were no significant differences between groups.

One sample (CON) was not brought to the laboratory in time and is therefore missing. Number of training days in the crush prior to the trial day had no significant influence on PCC at 0 min. There were no significant differences between groups at any time point (Figure 3). At baseline, mean PCC (with lower and upper CI) were 2,892.9 (1,603.6 – 5,218.7) pg/ml in CON and 2,864.1 (1,587.6 – 5,218.7) pg/ml in PAIN. PCC slightly increased following stimulus 2 in both groups and decreased after 1.5 h. In CON, PCC were significantly lower at 4.5 h compared with baseline and 0 min (*p* = 0.0163 and *p* = 0.0396, respectively). Course of PCC concentrations are presented in Figure 3. Mean PCC concentrations are presented in Supplementary Table 2.

### 3.4 Correlation between plasma substance P and cortisol concentrations

Overall, there was a negative correlation between PSPC and PCC, which was significant (*p* < 0.001, rho = −0.24). In CON, there was a negative correlation with a trend for significance (*p* = 0.06, rho = −0.14). In PAIN, the negative correlation was significant (*p* < 0.001, rho = −0.33).

### 3.5 Behavioral scoring during painful and sham stimulation

Data for behavioral assessment during the electrical stimulation is missing in one animal (4, PAIN) due to technical issues with the camera. Frequencies of behavior during the 30 min did not differ significantly between groups except for “shaking of the hind legs” (median of 4 occurrences for CON and 18 occurrences for PAIN, *p* = 0.003) for and “ear movements” (median of 166 occurrences for CON and 144 occurrences for PAIN, *p* = 0.023) (Table 5).

**Table 5:** Median frequencies of behavioral parameters and activity data assessed in calves submitted to either a SHAM (CON, *n* = 12) or an electrical (PAIN, *n* = 12) stimulus to assess the influence of an electrical stimulus without tissue damage on plasma substance P and cortisol concentrations, as well as the behavior and the activity in calves.

Parameter	Group		*p*-values
	CON	PAIN	
Parameters assessed during painful/sham stimulation (number in 30 min)			
Ear movements	166	144	***p* = 0.023**
Movement of the head	4	1	*p* = 0.97
Kicking with hind limbs	7	14	*p* = 0.19
Shaking of hind limbs	4	18	***p* = 0.003**
Tail movements	22.	30	*p* = 0.31
Moving forwards and backwards in crush	11	10	*p* = 0.73
Urination	2	3	*p* = 0.43
Activity parameters assessed over 24 h			
Lying time (minutes)	1,030 ± 54	1,027 ± 57	0.90
Downs (number)	25 ± 5	24 ± 6	0.91
Standing time (minutes)	402 ± 54	403 ± 59	0.96
Walking time (minutes)	9 ± 3	11 ± 4	0.19
Strides (number)	192 ± 61	227 ± 87	0.28
Activity changes (number)	123 ± 30	144 ± 46	0.21

Changes of behavior were assessed for 30 min during the stimulation and at each sampling point. Differences of vocalization, teeth grinding, and defecation could only be described descriptively due to the low numbers of presentation within the groups. The Mann–Whitney U test produced *p*-values based on rank comparisons, while medians are presented in the table for better understanding. Activity was assessed using pedometers for 24 h and is missing in one animal in CON due to technical problems. Significant differences between groups are indicated in bold letters.

### 3.6 Vital signs

RR is missing for 12 timepoints due to animals sniffling and investigators not being able to count a RR. At baseline, mean HR (lower and upper CI) was 115 (102.4–127) beats/min in CON and 116 (103.5–128) beats/min in PAIN, and mean RR (with lower and upper CI) was 34.9 (29.2–40.5) breaths/min for CON and 37.7 (32.1–43.4) breaths/min for PAIN. Mean frequencies of HR and RR are presented in Table 6.

**Table 6:** Mean values and lower and upper confidence intervals (CI) of heart rate and respiratory rate in *n* = 24 calves of the German Simmental Breed, which were either submitted to a sham (CON, *n* = 12) or an electrical (PAIN, *n* = 12) stimulus (indicated by box).

Time point	Heart rate (beats/min)			Respiratory rate (breaths/min)		
	CON	PAIN	*p*	CON	PAIN	*p*
Baseline	115 (102.4–127)	116 (103.5–128)	0.907	34.9 (29.2–40.5)	37.7 (32.1–43.4)	0.477
0 min	109 (96.6–121)	115 (103–127)	0.472	36.6 (31–42.2)	39.3 (33.6–45.0)	0.515
5 min	111 (99.1–124)	119 (106.5–131)	0.402	36.5 (30.9–42.1)	40.1 (34.4–45.7)	0.383
10 min	105 (92.9–117)	119 (106.9–131)	0.113	36.0 (30.4–41.6)	37.9 (32.1–43.8)	0.639
15 min	107 (94.8–119)	116 (104.1–129)	0.291	33.5 (27.9–39.1)	42.8* (37.2–48.4)	**0.022**
20 min	104 (91.8–116)	113 (100.6–125)	0.317	36.9 (31.3–42.5)	39.7 (34.1–45.3)	0.488
25 min	106 (93.4–118)	116 (104–128)	0.228	35.7 (30.1–41.4)	41.9 (36.2–47.7)	0.130
30 min	107 (94.8–119)	111 (98.7–123)	0.655	37.7 (32.1–43.3)	39.0 (33.4–44.6)	0.758
35 min	156**,^♦♦^ (143.5–168)	150**,^♦♦^ (138.2–163)	0.548	46.2**,^♦♦^ (40.5–52.0)	47.7**,^♦^ (42–53.5)	0.717
45 min	115 (102.6–127)	125 (112.7–137)	0.254	39.3 (33.6–45.0)	40.7 (35–46.5)	0.732
1 h	116 (103.6–128)	114 (101.8–126)	0.838	32.9 (27.2–38.6)	39.4 (33.6–45.1)	0.118
1.5 h	104 (91.8–116)	115 (102.6–127)	0.220	35.0 (29.4–40.7)	38.0 (32.4–43.7)	0.460
2 h	101* (88.9–113)	108 (96.1–121)	0.412	34.6 (29.0–40.2)	37.0 (31.4–42.7)	0.543
3 h	104* (91.4–116)	109 (96.4–121)	0.564	36.0 (30.4–41.6)	37.2 (31.5–42.8)	0.777
4.5 h	114 (101.6–126)	115 (102.6–127)	0.914	34.3 (28.7–40.0)	38.9 (33.2–44.5)	0.265
7 h	108 (95.9–120)	116 (104–128)	0.359	34.1 (28.5–39.7)	43.0 (37.1–48.8)	**0.031**

Significances (*p*) within groups are indicated, compared with baseline at 60 min (min) before intervention (* *p* < 0.05, ** *p* < 0.01) and at 0 min (start of intervention) (♦ *p* < 0.05, ♦♦ *p* < 0.01). Significant differences between groups are indicated in bold letters.

HR did not differ significantly between groups and was significantly higher at 35 min compared with baseline (*p* < 0.0001) and 0 min (*p* < 0.0001) both for CON and PAIN.

RR was significantly higher in PAIN compared with CON at 15 min (stimulus 3, 33.5 breaths/min for CON and 42.8 breaths/min for PAIN, *p* = 0.022) and 7 h (34.1 breaths/min for CON and 43.0 breaths/min for PAIN, *p* = 0.031). RR increased significantly at 15 min (stimulus 3) compared with baseline (*p* = 0.048) in PAIN. RR increased significantly at 35 min compared with baseline (*p* < 0.0001) both for CON and PAIN, and compared with 0 min (*p* < 0.0002 for CON and *p* = 0.016 for PAIN). Further significant differences within CON and PAIN for HR and RR are given in Supplementary Table 4.

### 3.7 Behavioral scoring at times of blood sampling

For “activity,” there were no occurrences of “does not look at observer, head down, ears back” at any time point before, during, and after stimulation, and no significant differences between the other two parameters between groups. There were no differences in “position of the head” between groups before and after stimulation. During stimulation, calves in CON showed significantly less occurrences of “head held below dorsal line” compared with calves in PAIN (*n* = 4 occurrences for CON and *n* = 6 occurrences for PAIN, *p* = 0.0406).

For “dorsal line of the back,” there were no occurrences of “mildy arched” or “severely arched,” but only for “straight, physiologic” in both groups before, during, and after stimulation.

Regarding the expression of the eyes, there were no occurrences for any parameter except for “normal, wide eyes” and “lids half closed,” which did not differ significantly between groups before stimulation. Calves in CON showed significantly more occurrences of “normal, wide eyes” during (*n* = 54 occurrences for CON and *n* = 43 occurrences for PAIN, *p* = 0.0141) and after (*n* = 98 occurrences for CON and *n* = 86 occurrences for PAIN, *p* = 0.0246) stimulation, whereas calves in PAIN showed significantly more occurrences of “lids half closed” during (*n* = 5 occurrences for CON and *n* = 17 occurrences for PAIN, *p* = 0.00743) and after (*n* = 8 occurrences for CON and *n* = 21 occurrences for PAIN, *p* = 0.0122) stimulation. Both calves of CON and PAIN showed a “clean, wet nose” at all time points before, during, and after stimulation. Occurrences of position and movements of the ears did not differ significantly between groups before, during, and after stimulation.

### 3.8 Activity and number of steps

Activity data is missing in one animal due to technical issues with the pedometer. Activity data and number of steps is given in Table 5. There were no significant differences between activity parameters between CON and PAIN.

## 4 Discussion

According to our findings, PSPC decreased following an electrical stimulus, with no significant changes to PCC, and only mild behavioral reactions. Therefore, our hypothesis that an electrical stimulus increases PSPC has to be rejected. To the best of our knowledge, this study is the first to present changes to PSPC during and following an electrical stimulation, which is the major strength of this study.

We used an electrical impulse for an electrical nociceptive stimulation as was done before in cattle (27), to reduce the risk of tissue damage or inflammatory processes influencing the PSPC, as would have been possible following a thermal, chemical, or mechanical stimulus. As electrical stimulation is noninvasive (31), it was the best option for the purpose of this study.

Electrical stimulation is not a natural type of stimulus. It excites all peripheral fibers, including large diameter fibers which are not directly involved in nociception. The electrical thresholds of these individual fibers are related to their diameters. This results in a stimulation of first Aβ-, followed by Aδ- and C-fibers with increased intensity of the stimulus. Therefore, electrical stimulation of a sensory nerve results in pain due to nonselective activation of all types of peripheral fibers (31). Contrary to electrical stimulation, thermal stimulation excites thermosensitive and nociceptive fibers. Mechanical stimulation activates low-threshold mechanoreceptors and nociceptors, and result in tissue damage if they are truly nociceptive (31).

Electrical stimulation was done every 5 min over a duration of 25 min (5 times), to imitate the continuous nociceptive stimulation of a painful surgery (28). Evasive behavior during stimulation could have been provoked by nociception, which is the physiologic process leading to pain perception (19), or by pain, which is defined as an unpleasant sensory and emotional experience associated with actual or potential tissue damage (19). However, the withdrawal response or reflex, an automatic response of the spinal cord to protect the body from harmful stimulation from noxious stimuli (32) could also have resulted in the evasive behavior (shaking of hind limb) shown by calves. Electrical stimulation was terminated when calves showed obvious signs of pain (shaking of the leg). After removal of the electrodes, following adspection and palpation, no tissue damage or alterations were recorded macroscopically, similar to adult cattle where only a hyperemia was recorded (27). As no biopsies were taken for histological examination, tissue damage cannot be ruled out microscopically, which is a limitation.

Even though calves in PAIN showed altered behavior indicative of pain, such as shaking and withdrawal of the leg (27) as well as half closed lids and head held below the dorsal line (3), PSPC did not differ between groups. PSPC decreased during the painful stimulus with the lowest concentrations at 25 min (last stimulus), and an increase after that stimulus. These findings are not in accordance with previous literature about PSPC in cattle exposed to nociception, where an increase in PSPC was found (6, 22), but similar to a recently published study, which found a decrease of PSPC during a stressful stimulation (23). Also, our findings are somewhat comparable to a previous study in bulls, which described that PSPC following electroejaculation (77.2 ± 17.2 pg./mL) did not differ from probed (79.1 ± 17.2 pg./mL) and control (93.4 ± 17.2 pg./mL) bulls. The authors concluded that electroejaculation did not result in nociception (33). Other studies only assessed PSPC in calves submitted to long lasting nociception in combination with the destruction of tissue (6, 7). Electrical stimulation is not a natural stimulus, but excites peripheral fibers, which are not directly involved in nociception (31), contrary to mechanical, chemical, or thermal stimuli which induce action potentials via nociceptors (19). SP is reported to be released following electrical stimulation in rats (34). However, transcutaneous electrical stimulation in rats supposedly reduces the production of SP in the dorsal root ganglion, resulting in analgesic effects by suppression of nociception via the C-fibers in the peripheral nerves (35). Research in cats showed that a single electrical stimulation of C-fibers was not mediated by NK-1 receptors, indicating that SP release is only mediated by specific patterns of firing nociceptors. This was further confirmed by the stimulation not being blocked by a NK-1 antagonist, suggesting that the response to the stimulus is not mediated by SP (36). Transcutaneous electrical nerve stimulation was found to inhibit the up-regulation of SP following skin/muscle incision and retraction in rats (37, 38). These findings could explain the absence of an increase of SP in our study population following electrical stimulation. However, a differentiation between procedures involving tissue damage, such as castration (6), inflammation such as due to lameness (22), or no tissue damage during electroejaculation (33) or a stressful stimulation (23) should be made regarding the evaluation of PSPC. It is possible that tissue damage, as well as inflammation is needed to raise PSPC. Therefore, PSPC might not be a good biomarker for nociception in the model used in the present study.

It is recommended to simultaneously determine PSPC and PCC to differentiate between acute stress due to handling and distress due to nociception (6, 7). It was found that the peak volume of PCC was reached 10 min after cattle being stressed by social separation (39). In the present study, there were no significant differences between groups, indicating that exposure to stress was the same for both groups. All animals had at least seven (8 to 15) days after purchase for acclimatization at the clinic and were trained to stand in the crush for 5 to 7 days. The acclimatization rate that it took heifers to move through a novel funnel structure in a yard was found to be decreased on days 1 and 2, and then consistent and increased on days 3 and 4 of training, respectively (40). Therefore, acclimatization and training period should have been sufficient for the present study. Different factors, such as restraint or presence of humans (7), as well as management and external environmental factors (41) can influence PCC. Significant differences within groups can be explained by calves being used to the procedure of the trial and of blood sampling at the end of the trial, resulting in less stress.

There was a negative and significant correlation between PSPC and PCC overall and in PAIN, which is not in accordance with previous findings in cattle which found a positive correlation between PSPC and PCC (23, 42). As a recently published study also found a significant decrease of PSPC in stressed calves but a positive correlation between PSPC and PPC (23), our findings could indicate that nociception and stress result in different interactions of cortisol and SP.

The observed behavioral responses are hard to compare with other studies, as previous research describing pain scales for cattle were working with a score system and defined thresholds or cut-off values associated with clinically relevant pain (3, 43). A grimace scale for pain and stress developed for calves also refers to a scoring system from scores zero to two (44). Therefore, an indication as to whether the observed behavioral responses reached thresholds associated with clinically relevant pain in calves cannot be made. As the stimulation calves were submitted to was in the context of a controlled trial and was limited until the moment calves showed continuous evasive behavior, it is possible that behavioral changes are more consistent with avoidance behavior than with nociception of sustained pain.

During stimulation, shaking of the stimulated hind leg was done significantly more often by PAIN calves and was the most frequent sign for discomfort and distress resulting in termination of the electrical stimulus. Seconds until termination decreased with significant differences from stimulus 1 to stimulus 4 and 5. Therefore, sensitization, which means that nociceptors are activated or become hypersensitive to subsequent stimulation (19, 45), cannot be ruled out. Shaking of the leg was not shown immediately following the start of the electrical stimulus as would have been expected in case of a withdrawal reflex, and calves often expressed stronger reactions to the subsequent stimuli and in some cases stereotypical behavior such as licking their halter, indicating that calves showed a conscious avoidance reaction. Frequency of occurrence of other parameters did not differ significantly, which can be explained by these behavioral patterns not being as specific for nociception as “shaking of the hind legs” in our study setup and is in accordance with a previous study investigating electric shocks in steers for vocalization, movements of the tail, and moving forwards and backwards in the crush (46). “Kicking with hind limbs” was mostly seen after the last electrical stimulus when the fixomull and the electrodes were removed from the skin and was therefore also recorded both in PAIN and CON. Contrary to previous findings describing more frequent head tossing in steers submitted to an electric shock compared to a control group (46), there was no difference in movements of the head between PAIN and CON in our study. This difference can be explained by the electrodes being placed behind the poll in those steers (46), and above the coronary band in the calves included in the present study. Behavioral reactions to the electrical stimulation were mild, contrary to an experiment in cattle which was terminated due to the severity of the behavioral responses of the animals (47). However, we stopped the electrical stimulation as soon as animals showed a continuous evasive behavior. It is possible that reactions would have been stronger and more severe if the electrical stimulation had not been stopped but continued until the end after 60 s. In cattle, the severity of behavioral responses was found to increase with the intensity of the electric shock (47). A study investigating the effect of an electrical prod on behavior in weaned beef cattle stated that beef cattle which had been touched, buzzed, and shocked with the electrical prod showed more escape reactions (such as running, kicking, or pushing through a block) compared with cattle that had only been touched or touched and buzzed (48). As the voltage in our study was continuously increased until a maximum of 60 s as opposed to a shock with a duration of 1 s (48), a comparison is hard to make. It should also be noted that the electrical stimulation used for the present study was not intended to model commercial handling procedures, but to serve as a controlled experimental tool. To limit the influence of investigators on behavior (49), the same number of people was always present during the period of the electrical or sham stimulation. Due to the setup of the study, the observer was not blinded to the grouping of the animal as recommended (49), which is a limitation. Also, interobserver reliability was not investigated, as behavioral assessment was either always done by TT (videos), or TT supervising HK.

Behavioral parameters assessed prior to every blood sampling did not differ between groups before, during, and after stimulation, except for the position of the head during, and expression of the eyes during and after the stimulation, with a higher occurrence of “lids half closed” in PAIN calves during and after the stimulation. The facial expression of animals changes when submitted to painful stimulation (50) and the facial grimace scale has been used for pain assessment in previous studies in cattle (4). The higher number of animals expressing “lids half closed” could be indicative of calves in PAIN experiencing nociception or pain during the electrical stimulation, even if the other parameters (ears, facial muscles, nuzzle) (3) did not differ between groups.

HR was significantly higher at 35 min compared with baseline in both groups, but did not increase following the electrical stimulation, as was also found for ten minutes following an electric shock in steers (46). Contrary to our findings, the heart rate of cattle increased monotonically immediately following an electrical shock in another study (47). The assessment of RR is an indirect measure to evaluate pain, even if not a satisfactory one (51). A previous study found no differences in RR between treatment groups in cattle submitted to surgery for left displaced abomasum with or without ketoprofen treatment (52). Significant differences in HR and RR between baseline and at 0 min compared with 35 min in both groups can be explained by walking the calves from the trial room to their igloos. Other factors, such as temperature humidity index, as well as posture of animals, seem to have an influence on RR (53), limiting the suitability of RR as an indicator for pain (52).

As electrical stimulation in PAIN was done above the coronary band of the right hind limb, changes to activity and steps taken due to pain should have been recorded. Pedometers directly record the locomotion and are therefore valuable for the assessment of lameness (54) and musculoskeletal pain (55). Similar to a previous study in calves undergoing tail docking (29), downs did not differ between CON and PAIN. Number of steps in our calves was similar with the baseline values of calves prior to tail docking housed under similar conditions. Contrary to our findings, number of steps was found to decrease within 1 h following tail docking (29), as well as following experimentally induced lameness in calves (54). The fact that there was no lameness in any calf or any differences between groups indicates that the electrical stimulation was not strong enough to result in tissue damage or long-lasting pain. However, limited room for movement restricts the evaluation of the “walking time.”

Calves weight was between 68 and 88 kg on the day of purchase. Body weight was not assessed on the day of either electrical or sham stimulation. A study showed that women’s tolerance to endure electrical muscle stimulation positively correlated with body weight, body fat mass, visceral fat area, and hip circumference (56). As applicators were placed around women’s abdomen (56), and the electrodes in the present study were placed above the coronary band of the right hind leg, where no large muscle or fat tissue can be found, an influence of body weight on our results seems unlikely. As cortisol and body weight are not correlated (57), body weight should not have had an effect on the PCC in the present study population.

To the best of our knowledge, this study is the first to present changes to PSPC during and following a noninvasive electrical stimulation, which is the major strength of this study, and to evaluate these changes in German Simmental calves.

One limitation of the present study is the low number of animals. The present study was conducted as a pilot study to assess the influence of an electrical stimulation on PSPC. A follow up study with a larger number of animals and comparison of different nociceptive stimuli, such as mechanical, thermal, or chemical should follow this study to further assess the suitability for SP as a biomarker for either nociception itself, or rather nociception as well as inflammation due to tissue damage.

Another limitation is the fact that calves needed to be clinically healthy on the day before and of the trial. Calves were submitted to a clinical examination prior to buying, but as calves were bought at a market, exposure to diseases was increased (58). Diarrhea and respiratory disease are the most common infectious diseases in calves (59), which was also the case in the present study population. As previous research showed that leucocyte count is significantly and positively related to PSPC in calves (20), calves were only included in the present study if leucocyte count on the day prior to the trial was within the clinic’s reference ranges to reduce the influence of inflammatory processes on PSPC. Calves were also excluded from the study if they had been treated either with an antibiotic or an inflammatory agent within 48 h prior to the trial, to limit the influence of diseases on the study results, and as the administration of analgesics results in a decrease of PSPC (14, 15).

The present study shows that a repeated short electrical stimulation does not increase, but continuously decrease PSPC in calves during the stimulation, even if animals show behavioral expressions of discomfort. It should be considered that, even if calves were clinically healthy and leucocyte count was within the reference ranges on the day before the trial, and animals had been trained to get used to the conditions of the trial, influence of pre-existing health conditions and stress on our study results, as well as of environmental factors, cannot be completely ruled out. As our findings are in contrast with other studies reporting an increase in PSPC following painful procedures in cattle, the authors could not assess if the evaluation of PSPC is a suitable method for assessment of noninvasive nociception in cattle which can be used individually and without relying on other parameters. Evaluation of PSPC may be a good marker for nociception in combination with tissue damage or inflammation but does not seem to be a reliable biomarker for nociception without tissue damage or inflammation. Studies to assess the influence of different nociceptive stimuli, such as mechanical, thermal, and chemical, with a larger number of animals will follow this study to establish basic research work regarding PSPC in the bovine.

## Data Availability

The raw data supporting the conclusions of this article will be made available by the authors, without undue reservation.
