# Supplementary material for: Influence of a repeated peripheral electrical stimulation on substance P and cortisol concentrations, and behavior in German Simmental calves — a pilot study
Source: Front Vet Sci. 2026 Jan 30;13:1752497. doi: 10.3389/fvets.2026.1752497 (PMC12902954; doi:10.3389/fvets.2026.1752497)
Supplement: Supplementary file 1 [file Table_1.docx]

Supplementary Material

**Supplementary Table 1:** General findings, findings of clinical examination according to Dirksen et al. (1979), and laboratory findings of selected blood parameters in n = 24 male calves of the German Simmental Breed on the day before trial day. Parameters are presented as mean and standard deviation (SD). Ranges are given in brackets.

|  | **Total**  (n = 24) | **CON**  (n = 12) | **PAIN**  (n = 12) |
| --- | --- | --- | --- |
| **General Findings** | | | |
| **Age (d)** | 43.9 ± 2.0  (39 – 47) | 41.1 ± 2.0  (39 – 47) | 44.8 ± 2.0  (42 – 47) |
| **Days in Crush** | 6.3 ± 0.8  (5 – 7) | 6.3 ± 0.9  (5 – 7) | 6.3 ± 0.8  (5 – 7) |
| **Findings of Clinical Examination** | | | |
| **Temperature (°C)** | 38.5 ± 0.4  (38.0 – 39.1) | 38.5 ± 0.4  (38.0 – 39.1) | 38.5 ± 0.4  (37.7 – 38.9) |
| **Heart Rate (beats/minute)** | 113.2 ± 10.4  (96 ± 140) | 114.7 ± 12.1  (100 – 140) | 111.7 – 8.8  (96 – 124) |
| **Respiratory Rate (breaths/minute)** | 38.3 ± 5.1  (28 – 48) | 36.7 ± 4.1  (28 – 44) | 40.0 ± 5.7  (28 – 48) |
| **Findings of Laboratory Analysis** | | | |
| **PCV^1^**  (30 – 36%) | 25.2 ± 5.5  (16.31 ± 36.68) | 26.5 ± 4.9  (18.41 – 36.68) | 23.9 – 5.9  (16.31 – 33.72) |
| **Leucocytes**  (4 – 10 x10³/µl) | 5.9 ± 1.2  (4.2 – 9.91) | 6.3 ± 1.4  (4.35 – 9.91) | 5.7 ± 1.0  (4.20 – 8.03) |
| **Total Protein**  (60 – 80 g/L) | 52.7 ± 4.3  (42.4 – 59.4) | 51.6 ± 5.0  (42.4 – 59.4) | 53.8 ± 3.3  (47.3 – 58.5) |
| **Albumin**  (30 – 40 g/L) | 33.3 ± 1.8  (29.9 – 37.4) | 32.6 ± 1.8  (29.9 – 35.2) | 34.0 ± 1.5  (31.8 – 37.4) |
| **Globulin**  (10 – 40 g/L) | 19.5 ± 3.6  (12.5 – 27.0) | 19.2 ± 4.4  (12.5– 27.0) | 19.7 ± 2.7  (7.4 – 12.6) |
| **Glucose**  (2.5 – 3.3 mmol/L) | 10.2 ± 2.2  (6.7 – 14.1) | 9.9 ± 2.5  (6.7 – 14.1) | 10.5 ± 1.9  (7.4 – 12.6) |

^1^Packed Cell Volume

**Supplementary Table 2:** Mean values and lower and upper confidence intervals (**CI**) of substance P and cortisol concentrations in n = 24 calves of the German Simmental Breed, which were either submitted to a sham (CON, n = 12) or an electrical (PAIN, n = 12) stimulus (indicated by box). Significances (**p**) within groups are indicated, compared with baseline 60 min before intervention (* p < 0.05, ** p < 0.01) and at 0 min (♦ p < 0.05, ♦♦ p < 0.01). There were no significant differences between groups for all laboratory parameters.

|  | **Substance P (pg/ml)** | | | **Cortisol (pg/ml)** | | |
| --- | --- | --- | --- | --- | --- | --- |
| **Time Point** | **CON** | **PAIN** | **p** | **CON** | **PAIN** | **p** |
| Baseline | 1,465.6  (880.1-2,440.6) | 1,685.8  (1,012.3-2,807.4) | 0.6981 | 2,892.9  (1,603.6-5,218.7) | 2,864.1  (1,587.6-5,218.7) | 0.9867 |
| 0 min | 1,495.2  (897.8-1,489.9) | 1,652.4  (992.3-2,751.8) | 0.7781 | 2,670.4  (1,480.3-4,817.4) | 2,864.1  (1,587.6-5,218.7) | 0.8539 |
| 5 min | 1,436.6  (854.1-2,392.3) | 1,495.2  (888.9-2,489.9) | 0.9136 | 2,835.6  (1,571.8-4,675.1) | 3,229.2  (1,790.1-5,884.0) | 0.7439 |
| 10 min | 1,380.2  (828.8-2,298.5) | 1,636.0  (982.4-2,2724.4) | 0.6436 | 3,294.5  (1,808.0-6,002.9) | 3,789.5  (2,100.6-6,836.3) | 0.7434 |
| 15 min | 1,212.0  (720.5-2,018.3) | 1,556.2  (934.5-1,591.5) | 0.4783 | 3,568.9  (1,978.3-6,438.2) | 3,197.1  (1,772.2-5,767.5) | 0.7886 |
| 20 min | 1,465.6  (871.3-2.446.6) | 1,176.1  (706.3-1,958.6) | 0.5516 | 3,197.1  (1,722.2-5,767.5) | 3,789.5  (2,100.6-6,836.3) | 0.6930 |
| 25 min | 1,299.8  (780.6-2,164.6) | 962.9*,^♦^  (572.5-1,603.6) | 0.4053 | 2,835.6  (1,571.8-5,115.3) | 3,133.8  (1,737.1-5,653.3) | 0.8063 |
| 30 min | 1,366.5  (820.6-2,275.6) | 1,176.1  (706.3-1,958.6) | 0.6812 | 3,010.9  (1,652.4-5,486.2) | 3,071.7  (1,702.8-5,541.4) | 0.9591 |
| 35 min | 1,286.9  (765.1-2,143.1) | 1,636.0  (972.6-2,724.4) | 0.5095 | 3,394.8  (1,881.8-6,124.2) | 3,641.0  (2,018.3-6,568.2) | 0.8588 |
| 45 min | 1,540.7  (925.2-2,591.5) | 1,587.6  (953.4-2,643.9) | 0.9369 | 3,568.9  (1,978.3-6,438.2) | 3,463.4  (1,900.0-6,247.9) | 0.9309 |
| 1 hour | 1,436.6  (854.1-2,392.4) | 1,236.5  (735.1-2,059.1) | 0.6791 | 3.394,8  (1.881.8-6,124.2) | 4,402.8  (2,440.6-8,022.5) | 0.5312 |
| 2 hours | 1,199.9  (720.5-1,998.2) | 1.754.6  (1,053.6-2,921.9) | 0.2936 | 2,079.7  (1,152.9-3,751.8) | 2,368.5  (1,312.9-4,315.6) | 0.7497 |
| 3 hours | 1,540.7  (925.2-2,565.7) | 1,556.2  (934.5-2,591.5) | 0.9713 | 2,143.1  (1,176.1-3,866.1) | 2,489.9  (1,380.2-4,491.8) | 0.7093 |
| 4.5 hours | 1,587.8  (953.4-2,643.9) | 1,286.9  (772.8-2,143.1) | 0.5612 | 1,636.0***,^♦^**  (906.9-2,951.3) | 3,041.2  (1,685.8-5,486.2) | 0.1418 |
| 7 hours | 1,394.1  (828.8-2,344.9) | 1,465.6  (880.1-2,440.6) | 0.8974 | 2,565.7  (1,408.1-4,675.1) | 2,121.8  (1,176.1-3,866.1) | 0.6671 |

**Supplementary Table 3**: Significant differences within groups for plasma substance P concentrations in n = 24 calves of the German Simmental Breed, which were either submitted to a sham (CON, n = 12) or an electrical (PAIN, n = 12) stimulus. In CON, there were no significant differences within the group.

| **PSPC (pg/ml)** | | | | | |
| --- | --- | --- | --- | --- | --- |
| **CON** | | | **PAIN** | | |
| **Timepoint** | | **P** | **Timepoint** | | **P** |
|  |  |  | Baseline | 25 min | 0.0057 |
|  |  |  | 0 min | 25 min | 0.0075 |
|  |  |  | 5 min | 25 min | 0.0301 |
|  |  |  | 10 min | 25 min | 0.0087 |
|  |  |  | 15 min | 25 min | 0.0167 |
|  |  |  | 20 min | 2 hours | 0.0486 |
|  |  |  | 25 min | 35 min | 0.0092 |
|  |  |  | 25 min | 45 min | 0.0129 |
|  |  |  | 25 min | 2 hours | 0.0030 |
|  |  |  | 30 min | 2 hours | 0.0488 |

**Supplementary Table 4:** Significant differences (**p**) of heart rate, and respiratory rate between time points within two different groups of calves, which were either submitted to a sham (CON, n = 12) or an electrical (PAIN, n = 12) stimulus (from 5 min to 25 min). Abbreviations: BL = Baseline; minute = min; hour(s) = h.

| **Heart Rate (beats/minute)** | | | | | | **Respiratory Rate (breath/minute)** | | | | | |
| --- | --- | --- | --- | --- | --- | --- | --- | --- | --- | --- | --- |
| **CON** | | | **PAIN** | | | **CON** | | | **PAIN** | | |
| **Timepoint** | | **P** | **Timepoint** | | **P** | **Timepoint** | | **P** | **Timepoint** | | **P** |
| BL | 35 min | <0.0001 | BL | 35 min | <0.0001 | BL | 35 min | <0.0001 | BL | 35 min | 0.0001 |
| BL | 2 h | 0.0165 | 0 min | 35 min | <0.0001 | 0 min | 35 min | 0.0002 | 0 min | 35 min | 0.0016 |
| BL | 3 h | 0.0495 | 5 min | 35 min | <0.0001 | 5 min | 35 min | 0.0002 | 5 min | 35 min | 0.0034 |
| 0 min | 35 min | <0.0001 | 10 min | 35 min | <0.0001 | 10 min | 35 min | 0.0001 | 10 min | 35 min | 0.0004 |
| 5 min | 35 min | <0.0001 | 15 min | 35 min | <0.0001 | 15 min | 35 min | <0.0001 | 15 min | 2 h | 0.0244 |
| 10 min | 35 min | <0.0001 | 20 min | 35 min | <0.0001 | 15 min | 45 min | 0.0268 | 15 min | 3 h | 0.0275 |
| 15 min | 35 min | <0.0001 | 20 min | 45 min | 0.0319 | 20 min | 35 min | 0.0004 | 20 min | 35 min | 0.0022 |
| 20 min | 35 min | <0.0001 | 25 min | 35 min | <0.0001 | 25 min | 35 min | 0.0001 | 25 min | 35 min | 0.0308 |
| 20 min | 1 h | 0.0355 | 30 min | 35 min | <0.0001 | 30 min | 35 min | 0.0012 | 30 min | 35 min | 0.0008 |
| 25 min | 35 min | <0.0001 | 30 min | 45 min | 0.0132 | 35 min | 45 min | 0.0099 | 35 min | 45 min | 0.0090 |
| 30 min | 35 min | <0.0001 | 35 min | 45 min | <0.0001 | 35 min | 1 h | <0.0001 | 35 min | 1 h | 0.0018 |
| 35 min | 45 min | <0.0001 | 35 min | 1 h | <0.0001 | 35 min | 1.5 h | <0.0001 | 35 min | 1.5 h | 0.0002 |
| 35 min | 1 h | <0.0001 | 35 min | 1.5 h | <0.0001 | 35 min | 2 h | <0.0001 | 35 min | 2 h | <0.0001 |
| 35 min | 1.5 h | <0.0001 | 35 min | 2 h | <0.0001 | 35 min | 3 h | 0.0001 | 35 min | 3 h | 0.0001 |
| 35 min | 2 h | <0.0001 | 35 min | 3 h | <0.0001 | 35 min | 4.5 h | <0.0001 | 35 min | 4.5 h | 0.0007 |
| 35 min | 3 h | <0.0001 | 35 min | 4.5 h | <0.0001 | 35 min | 7 h | <0.0001 | 2 h | 7 h | 0.0269 |
| 35 min | 4.5 h | <0.0001 | 35 min | 7 h | <0.0001 | 45 min | 1 h | 0.0167 | 3 h | 7 h | 0.0301 |
| 35 min | 7 h | <0.0001 | 45 min | 2 h | 0.0033 | 45 min | 7 h | 0.0458 |  |  |  |
| 45 min | 2 h | 0.0150 | 45 min | 3 h | 0.0039 |  |  |  |  |  |  |
| 45 min | 3 h | 0.0454 |  |  |  |  |  |  |  |  |  |
| 1 h | 1.5 h | 0.0357 |  |  |  |  |  |  |  |  |  |
| 1 h | 2 h | 0.0091 |  |  |  |  |  |  |  |  |  |
| 1 h | 3 h | 0.0295 |  |  |  |  |  |  |  |  |  |
| 2 h | 4.5 h | 0.0241 |  |  |  |  |  |  |  |  |  |
